# The prospective associations among time management tendency, negative emotions, and problematic smartphone use in Chinese nursing students: enlightenment from COVID-19

**DOI:** 10.3389/fpubh.2024.1323273

**Published:** 2024-02-08

**Authors:** Nani Ding, Jiaqi Shi, Huihui Xu, Xiaoyi Wang, Guilin Liu, Lijie Mao, Guohua Zhang, Jingjing Zhang

**Affiliations:** ^1^Wenzhou Vocational College of Science and Technology, Wenzhou, China; ^2^School of Nursing, Wenzhou Medical University, Wenzhou, China; ^3^Department of Psychology, School of Mental Health, Wenzhou Medical University, Wenzhou, China; ^4^School of Public Health and Management, Wenzhou Medical University, Wenzhou, China; ^5^First Affiliated Hospital of Wenzhou Medical University, Wenzhou, China; ^6^Zhejiang Provincial Clinical Research Center for Mental Disorders, The Affiliated Wenzhou Kangning Hospital, Wenzhou Medical University, Wenzhou, China

**Keywords:** nursing student, problematic smartphone use, time management tendency, negative emotions, COVID-19 pandemic

## Abstract

**Purpose:**

The regularity of epidemic prevention and control measures in China has meant that nursing students have been exposed to more electronic devices, while problematic smartphone use has increased. The purpose of this study is to determine the prospective associations among time management tendency, negative emotions, and problematic smartphone use in nursing students during the COVID-19 pandemic.

**Methods:**

A longitudinal study was conducted between November 2021 and May 2022. A total of 989 nursing students participated. The convenience sampling method was adopted and the following tools were used: the Adolescence Time Management Disposition Scale, the Depression Anxiety Stress Scales – 21, and the Mobile Phone Addiction Index. Multiple parallel mediation models were used by Mplus.

**Results:**

Time management tendency had a significantly negative effect on problematic smartphone use (*p* < 0.05). Further tests using mediation models showed that stress as a negative emotion mediated the relationship between time management tendency and problematic smartphone use (*p* < 0.05) over time.

**Conclusion:**

Nursing educators need to strengthen the stress resistance and time management ability of nursing students.

## 1 Introduction

In order to alleviate the impact of the rapid development of the COVID-19 on medical institutions, the Chinese government pursues a “soft landing” policy for epidemic prevention (1). Under this background, nursing students have been “home confinement” and “online teaching” for a long time. This has led nursing students to contact with smartphones for a long time, which is easy to lead to problematic smartphone use (PSU) (2, 3). According to the definition provided by the Diagnostic and Statistical Manual of Mental Disorders (DSM-V), the diagnosis of PSU includes a range of negative emotional syndromes (4), including excessive smartphone use by the user, and feelings of shame when leaving the phone scene. Globally, the prevalence of PSU in nursing students ranges from 9.3 to 33.1% (5). Excessive use of mobile phones reduces their learning and work ability, and impairs their interpersonal relationships (6, 7).

On December 13, 2022, China’s epidemic control was fully released, resulting in a large number of infected people in a short time in China and leading to a lag in the peak of infection. On the one hand, nursing students are worried about infection with COVID-19, and on the other hand, they are worried about the decline of their academic performance due to long-term online teaching. Under such conditions, the level of anxiety and depression of nursing students is higher than usual (8). Negative motions (such as anxiety, depression, and stress) may be one of the essential triggers of PSU in nursing students. Several studies have shown that individuals are at high risk of cell phone addiction when they experience anxiety, depression or stress from their environment (9, 10). The relationship between negative emotions and PSU among Chinese nursing students is even more pronounced. A cross-sectional study investigated 2,182 Chinese medical students and found that 39.7% had varying degrees of PSU, with mental health problems showing a significant association (11).

Secondly, nursing students have a lot of spare time due to long-term home-quarantined, which leads to low level of time management tendency (12). Time management tendency (TMC) refers to a range of behaviors that contribute to an individual’s achieving individual or organizational goals through their attitudes and values with regard to time (13). Good time management tendencies can promote the rational use of time by nursing students, a solid theoretical and practical foundation, and the development of good self-management and interpersonal skills (14, 15). Leisure time theory explains the vital role played by time management tendency. Individuals with low time management tendency are more likely to allocate leisure time to their smartphone, leading to PSU (16).

In addition, the negative emotion caused by the low level of TMC further aggravates the PSU. The Interaction of Person-Affect-Cognition-Execution (I-PACE) model (17) highlights the important role of personal factors in PSU. The model suggests that personal factors (e.g., personality, social cognition, genetics) can act as trigger variables that induce emotional and cognitive responses in specific situations, which then induce stress responses and act as stimuli for cell phone addiction and problematic smartphone use. For example, time management bias is a personality trait (18). Individuals with a low propensity for time management are prone to lifestyle disorders that affect productivity, increase work-study stress, and further negative emotions (19). To mitigate the effects of negative emotions, individuals focus more on short-term reward and risky decisions, increasing their propensity for using smartphones (20).

As of February 11, 2023, the Chinese government officially announced that the epidemic was basically over. However, the psychological trauma caused by the epidemic and the problematic mobile phone use caused by the low level of time management tendency are difficult to recover in a short time (21). This requires nursing educators to scientifically guide nursing students out of psychological difficulties, help them reduce their dependence on mobile phones and grow healthily. At the same time, the nursing teaching model needs to be systematized in order to deal with the unexpected major health events in the future. These measures are inseparable from data support.

**Figure 1:**
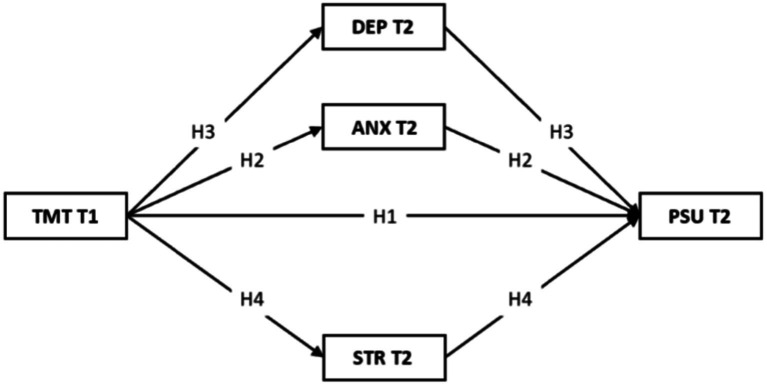
Research hypothesis. TMT, Time Management Tendency; PSU, Problematic Smartphone Use; DEP, Depression; ANX, Anxiety; STR, Stress.

Based on the I-PACE model, this paper presents a longitudinal study which was designed to explore the underlying mechanisms surrounding the effects of negative emotions (i.e., anxiety, depression, and stress) and time management tendency on problematic smartphone use. The hypotheses of the study are as follows (see Figure 1):

*Hypothesis 1*: TMT has a significant negative correlation with PSU over time.

*Hypothesis 2*: Anxiety will mediate the relationship between TMT and PSU over time.

*Hypothesis 3*: Depression will mediate the relationship between TMT and PSU over time.

*Hypothesis 4*: Stress will mediate the relationship between TMT and PSU over time.

## 2 Materials and methods

### 2.1 Participants and procedure

The participants in this longitudinal study were full time undergraduate nursing students at Wenzhou Medical University. The study was part of a university-based mental health follow-up program among Chinese undergraduates, and participation in the study was voluntary. Inclusion criteria: (1) Chinese Han nationality; (2) nursing college students; (3) no prior or co-existing mental illness, including schizophrenia, bipolar disorder, obsessive-compulsive disorder, psychoactive substance abuse or dependence, mental retardation, dementia, organic disease or drug-induced mental disorders; (4) No cognitive dysfunction, communication disorder, vision/hearing impairment and other people who could not successfully complete the questionnaire. Nursing students who were unable to participate in the study due to the quarantine policy were excluded from the study. The data were collected in November 2021 (Time 1, T1) and May 2022 (Time 2, T2). The sample size methodology for generalized multivariate analysis by KENDALL recommends taking a sample size that is 5 to 10 times the number of variables (22). Considering the 10% invalid response rate, we expanded the minimum sample size to *n* = 451. The study was approved by the Research Ethics Committee of the Wenzhou Medical University in China. All participants were informed of the study purpose and procedures and provided their informed consent to participate. Self-report questionnaires were distributed to all students during each wave through Wenjuanxing, an online crowd sourcing platform in China. Each participant’s ID (but not name) was recorded for matching the longitudinal data. The researchers were not able to access students’ names from their ID numbers, and the study was therefore anonymous. The researchers also vouched for the confidentiality of the data which would only be accessible to the researchers. Participants were provided with information on local professional help resources in case of need.

The data were gathered from 989 participants at baseline, of which 27 cases (2.73% of the 989) were excluded from data analyses because one or more scales of the major variables were rated in a particular way independently of the question content (e.g., selecting the same number on a Likert-type scale throughout for one or more scales). Subsequently, 938 participants (97.50% of the 962) remained in the study at the second wave after some were ruled out because of absenteeism or changes in majors. Data from the participants who completed all two surveys (n = 938, 85.3% female) were used for analysis.

### 2.2 Measures

#### 2.2.1 Adolescence time management disposition scale

The ATMD was developed by Huang and Zhang (23) and is suitable for college students. The ATMD consists of 44 items including the sense of time value subscale, the sense of time control subscale, and the sense of time efficacy subscale. The score for each item assigns 1 to 5 points from “completely inconsistent” to “completely consistent,” and the cumulative score for all dimensions is the total score of ATMD (range: 44–220). The higher the score, the better the time management skills. Through a strict psychometric process, the scale has good reliability and validity (24) and is suitable for evaluating Chinese college students’ time management skills. In the present study, the Cronbach’s α of the scale was 0.94 at T1.

#### 2.2.2 Depression anxiety stress scales – 21

The DASS–21 measures symptoms of depression, anxiety, and stress (25), comprising 21 questions. The questionnaire is divided into three subscales and each subscale has seven items: depression (DASS 21–D), anxiety (DASS 21–A), and stress (DASS 21–S). Each item is scored on a 4-point scale ranging from 0 (“does not apply to me at all”) to 3 (“applies to me very much”). The total score ranges from 0 to 63. The alpha coefficients for the reliability of the depression, anxiety and stress scales in Lu′s study were 0.82, 0.82, and 0.79, respectively (26). In the present study, the Cronbach’s α of the subscales were 0.90 (DASS 21–D), 0.94 (DASS 21–A), and 0.94 (DASS 21–S) at T2, respectively.

#### 2.2.3 Mobile phone addiction index

Problematic smartphone use was assessed using the MPAI (27). The MPAI consists of 17 items scored from 1 to 5 indicating “never” to “always.” Total scores range from 17 to 85. The diagnostic criteria of the scale are positive answers to more than 8 items, and higher scores indicating higher levels of problematic smartphone use. In Huang’s study, the Cronbach’s α of the scale was 0.92. In the present study, the Cronbach’s α was 0.92 at T2.

### 2.3 Data analysis

Descriptive and correlation analyses were performed using IBM SPSS 24.0. No missing data were evident in the final sample since consenting participants were prompted to complete all items. Pearson’s correlations were conducted to examine firstly the correlations between gender, time management tendency, depression, anxiety, stress, and problematic smartphone use. Then, considering the multifaceted nature of mental health mechanisms, the longitudinal multiple parallel mediation model was computed using Mplus (28, 29). In line with the study hypotheses, we proposed time management as an independent variable, problematic smartphone use as a dependent variable, and mental health (i.e., depression, anxiety, and stress) as parallel mediators of the relationships between variables (30). Since gender is a well-documented factor in mental health, especially for nursing undergraduates, it was adjusted for in the model. The level of statistical significance was 0.05.

## 3 Results

### 3.1 Descriptive findings

Harman’s single factor test was performed to test for common method bias, with 82 principal components extracted without rotation. The explanatory rate of the total variance variation of the first component was 21.2%, lower than the critical value of 40.0% (31). Thus, there was no serious common method bias in the data.

Table 1 provides descriptive statistics including the means, standard deviations, and the intercorrelations among study variables, which show significant correlations between time management tendency, depression, anxiety, stress, and problematic smartphone use. In terms of gender, only depression and anxiety were significantly and negatively correlated.

**Table 1:** Mean (M), standard deviation (SD) and Pearson correlations.

VARIABLES	*M* ± SD	1	2	3	4	5
1. Gender		1				
2. TMT T1	154.89 ± 19.26	0.04	1			
3. PSU T2	54.12 ± 10.92	0.03	−0.16***	1		
4. DEP T2	3.83 ± 4.51	−0.09**	−0.16***	0.40***	1	
5. ANX T2	4.25 ± 4.45	−0.07*	−0.14***	0.43***	0.93***	1
6. STR T2	4.60 ± 4.63	−0.05	−0.14***	0.44***	0.91***	0.93***

Male: 0, Female: 1; TMT, Time Management Tendency; PSU, Problematic smartphone Use; DEP, Depression; ANX, Anxiety; STR, Stress; *p < 0.05; **p < 0.01; ***p < 0.001.

### 3.2 Mediation results

**Figure 2:**
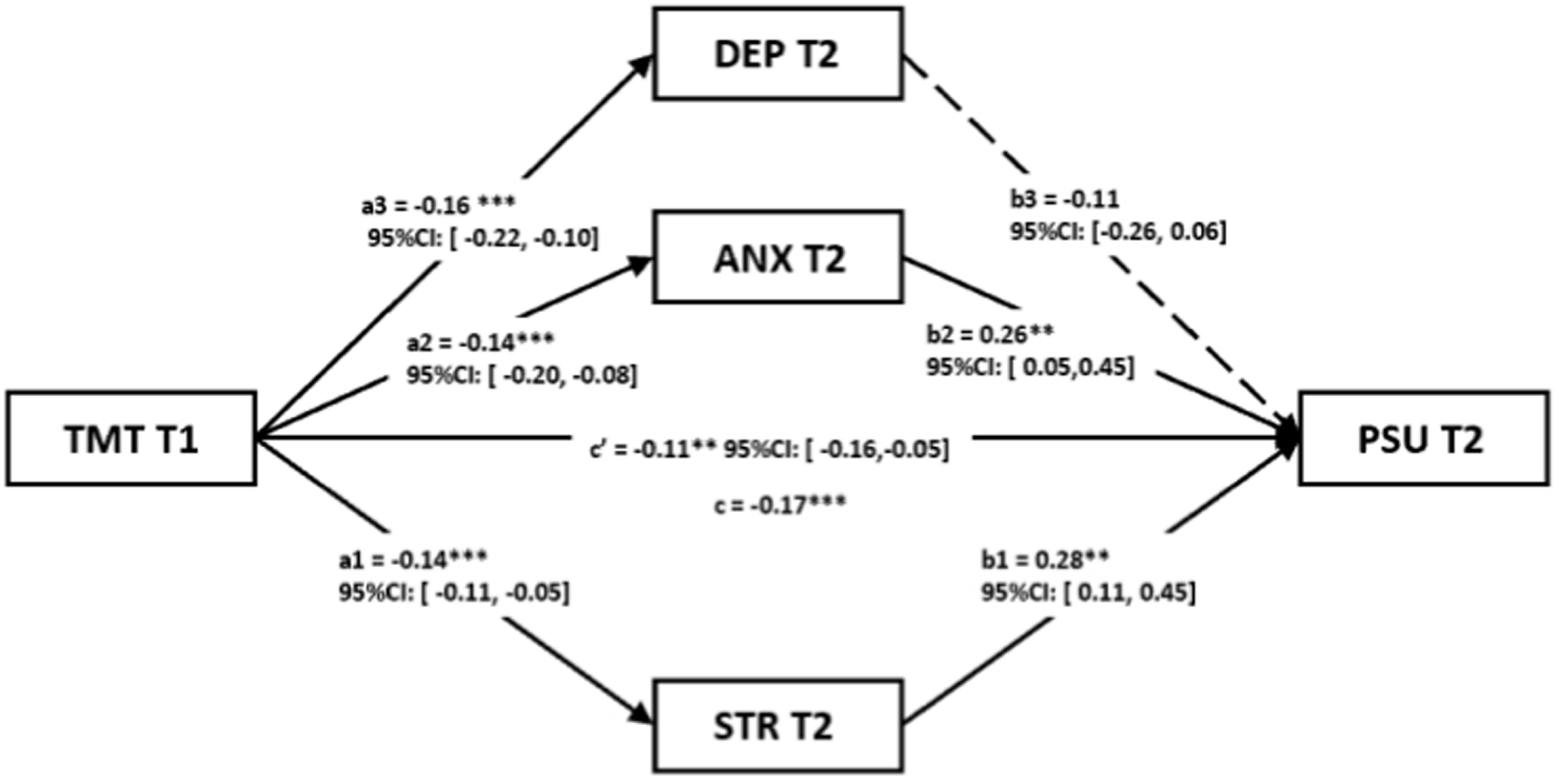
The longitudinal multiple parallel mediation model (*n* = 938). TMT, Time Management Tendency; PSU, Problematic Smartphone Use; DEP, Depression; ANX, Anxiety; STR, Stress; *p < 0.05; **p < 0.01; ***p < 0.001.

In order to eliminate the impact of gender on the results, we conducted a longitudinal multiple parallel mediation model analysis and presented the detailed results after controlling for gender (Figure 2). Overall, we found a significant total indirect effect [ab = −0.06; 95%CI: (−0.09, −0.04); *p* < 0.001] of negative emotions as a mediator between time management tendency and problematic smartphone use. In particular, stress at T2 [a1b1 = −0.04; 95%CI: (−0.08, −0.02); *p* = 0.037] showed a significant indirect effect on the relationship as previously mentioned. However, we could not replicate these findings considering levels of anxiety at T2 [a2b2 = −0.04; 95%CI: (−0.08, −0.01); *p* = 0.064] and depression at T2 [a3b3 = 0.02; 95%CI: (−0.01, 0.05); *p* = 0.276]. The direct effect of TMT on PSU was significant [c’ = −0.11; 95%CI: (−0.16, −0.05); *p* = 0.002]. Finally, we observed a significant total effect (c = −0.17; *p* < 0.001).

## 4 Discussion

Based on the I-PACE model, this study has explored the prospective relationships among time management tendency, negative emotions, and problematic smartphone use in Chinese nursing undergraduates during the COVID-19 pandemic. The results showed that in the COVID-19 context the low level of time management tendency was one of the leading contributors to nursing students’ problematic smartphone use, and stress played a mediating role between the two factors. In order to furnish theoretical underpinnings, it is imperative that we consolidate the management and pedagogical experiences during the COVID-19 pandemic, and establish a robust mechanism for the management of nursing students. This will enable us to scientifically regulate the usage of smartphone by nursing students, and effectively respond to major public health crises.

### 4.1 The prospective relationship between time management tendency and problematic smartphone use

The results support Hypothesis 1 that time management tendency is negatively associated with problematic smartphone use over time. The present finding suggests that nursing students with low time management tendency levels have poor self-regulation ability and find it difficult to resist the temptation brought by smartphones (6, 32). Altiner (33) hold the view that the low time management tendency of nursing students may be a fundamental reason for their inability to control the time spent on smartphones, which damages their academic performance and physical and mental health. According to the I-PACE model, time management tendency is thought to be an induced variable that represents a core human trait that induces problematic smartphone use. A good sense of time management enables nursing students to become more involved in their studies and lives and to use their smartphones rationally.

The findings of this study provide theoretical support for subsequent interventions: time management training can improve the mental health level and academic performance of nursing students (34), which should inspire educators to set up courses on efficient time management in medical schools to improve nursing students’ learning efficiency and productivity.

### 4.2 Mediation of negative emotions

The results also support Hypothesis 4 that time management tendency can reduce the incidence of problematic smartphone use in nursing students triggered by stress as a mediator. According to Elsey (35), early childhood exposure to stressors can lead to stronger responses to stressors in adolescents and adults that develop into addictive behaviors. Based on the I-PACE model, stress-prone individuals are more likely to regulate emotions in the face of stress, exacerbating cell phone addiction. For nursing students, the post-traumatic stress disorder received during COVID-19 is difficult to recover in the short term. Anxiety and depression still affect their studies and life (36). Nursing educators could carry out rich psychological and cultural activities through group sandbox games, psychological skits, and heart-to-heart conversations, which enhance nursing students’ ability to relieve negative emotions (37, 38). In addition, nursing educators should establish a psychological crisis intervention mechanism under public health events to prevent anxiety from damaging the physical and mental health of nursing students, and block this path to reduce the occurrence of problematic mobile phone use (39).

However, Hypothesis 2 and Hypothesis 3 were not supported by the results, which differs from the results of previous research (32, 40). This may be due to the study design and the choice of research tools. On the other hand, depression is a mood disorder characterized by a loss of interest in normal life. It is conceivable that depression as a negative symptom may lead to decreased interests and low moods, which causes an individual’s interest in exploring smartphones to decrease and blocks this pathway. Future studies that investigate further whether depression and anxiety influence problematic smartphone use could focus on transient emotions when nursing students use smartphones.

### 4.3 Enlightenment of nursing education

During the COVID-19 pandemic, nursing students were forced to adopt online courses, a non-traditional mode of learning. Although this maintained the continuity of education, it brought about side effects such as problematic smartphone use, negative emotions, and ineffective time management (41). Even though the impact of the pandemic is gradually waning and people are returning to their regular learning and working routines, we must reflect on these experiences, address these issues, and prepare for potential future public health challenges: 1. Online courses have led to students becoming more dependent on digital devices, especially smartphones. To prevent this dependency from turning into problematic smartphone use and affecting their studies, we need to develop learning platforms focused on mitigating distractions and promoting healthy usage. Educators should collaborate with developers to set time limits to regulate device usage appropriately (42). 2. An increase in negative emotions might reflect the isolation and need for learning support triggered by remote learning. To address this issue, we should establish and strengthen online learning communities, encourage mutual support among students, alleviate feelings of loneliness and negativity, and enhance motivation for learning (43). 3. Students might find it difficult to manage their time without the environment of face-to-face classes. Therefore, training in time management and self-discipline skills should be reinforced during the educational process. Additionally, regularly evaluating the effectiveness of online teaching and adjusting instructional plans based on student feedback can help tailor education services to better meet students’ actual needs and learning habits (44).

### 4.4 Limitations and future prospects

This longitudinal study has shed light on the underlying mechanisms of problematic smartphone use in nursing students. However, the research has the following limitations. First, considering that our sample is relatively homogenous, limited in scope, and mostly female, generalizations from the results should be cautious. Second, the results may have been influenced by social expectations or memory bias of the participants. Transient assessment tools may be considered in the future. Third, more follow-up studies are needed to reveal the causal relationships among the variables. Lastly, The novel coronavirus has now been classified as a Class B infectious disease by the Chinese government, indicating that isolation measures will no longer be implemented for those infected with the virus, nor will close contacts be identified. The teaching of nursing students in China has also gradually returned to normal, marking a significant milestone in our battle against the virus, it has provided theoretical support for future contingencies in response to unforeseeable public health crises. It is imperative that we reflect on the experiences and lessons learned in the management of nursing education during the COVID-19 pandemic, in order to prepare for any future challenges that may arise.

## 5 Conclusion

This study expanded our understanding of the longitudinal relationships among time management tendency, negative emotions, and problematic smartphone use during the COVID-19 pandemic among nursing students. The results indicated that nursing students with low time management levels are more likely to develop problematic smartphone use, while stress serves as a mediator between them. The findings implies that we should assist nursing educators in designing targeted interventions to reduce problematic smartphone use and stress, and to improve time management tendency.

## Data availability statement

The raw data supporting the conclusions of this article will be made available by the authors, without undue reservation.

## Ethics statement

This study was conducted following the Declaration of Helsinki. Informed consent was obtained from all subjects. Ethical approval was obtained from the Ethics Committee of Wenzhou Medical University: [2022-028].

## Author contributions

ND: Writing – review & editing. JS: Writing – original draft. HX: Formal analysis, Writing – original draft. XW: Investigation, Writing – original draft. GL: Software, Writing – original draft. LM: Data curation, Writing – original draft. GZ: Methodology, Writing – original draft. JZ: Supervision, Writing – original draft, Writing – review & editing.
